# The Effect of Electroconvulsive and Magnetic Seizure Therapy (MST) on Cortical Thickness in Schizophrenia

**DOI:** 10.1111/cns.70309

**Published:** 2025-03-03

**Authors:** Jin Li, Junjie Wang, Yong Yang, Ju Gao, Xiaobin Zhang

**Affiliations:** ^1^ Institute of Mental Health, Suzhou Psychiatric Hospital The Affiliated Guangji Hospital of Soochow University Suzhou Jiangsu China

**Keywords:** brain cortical thickness, ECT, MST, neuropsychological assessment, SCS

## Abstract

**Background:**

Identifying ways to conduct brain stimulation that match the clinical efficacy of electroconvulsive therapy (ECT) without the side effects of ECT is an important goal in schizophrenia (SCS). Magnetic seizure therapy (MST) is a potential alternative, which has shown considerable efficacy but with mild cognitive impairment.

**Objective:**

This study compared the clinical efficacy and cognitive side effects of ECT and MST. In addition, we also investigated the possible contribution of cortical thickness changes to treatment response.

**Methods:**

Thirty‐four confirmed schizophrenia patients were randomly treated with ECT (*n* = 16) or MST (*n* = 18) for 4 weeks. Mental symptoms were measured through PANSS, cognition was measured through the Repeatable Battery for the Assessment of Neuropsychological Status (RBANS), and changes in cortical thickness before and after treatment were compared using FreeSurfer.

**Results:**

Both treatments reduced the PANSS score and had comparable efficacy, while MST was superior in preserving the RBANS language score.

**Conclusion:**

In this study, neither the MST group nor the ECT group showed significant changes in cortical thickness after treatment. MST, like ECT, effectively alleviates symptoms of schizophrenia but retains cognitive function slightly better.

**Trial Registration:**

ClinicalTrials.gov identifier: NCT02746965

## Graphical abstract

Does magnetic shock have an impact on the brain? No, it is effective and safe! Magnetic shock therapy (MST) has significant advantages in treatment. After the patient was admitted, the doctor recommended more advanced magnetic shock therapy to her, which caused less damage to the brain's cognition and memory. The focus of this project is to explore the effect of magnetic shock on the thickness of the cerebral cortex.

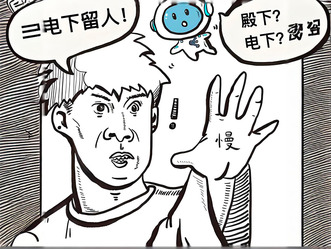

## 1 Introduction

Schizophrenia is a chronic and serious mental disease, which is mainly manifested in the abnormalities of sensation, perception, emotion, and behavior, and will have a serious impact on the lives of patients and their families [1]. Although antipsychotic drugs are one of the main choices for the management of schizophrenia, about one‐third of schizophrenics do not respond well to first or second‐generation antipsychotic drugs [2]. Electroconvulsive therapy (ECT) is a potential choice for patients with limited drug response [3].

Although electroconvulsive therapy (ECT) was effective and safe in the treatment of schizophrenia, the potential for cognitive impairment must always be carefully weighed [4]. Magnetic seizure therapy (MST) was the use of transcranial magnetic stimulation to induce seizures [5]. The magnetic field passes through the tissue unhindered and can control the stimulation site and scope better than ECT [6]. The clinical study of MST in the treatment of schizophrenia shows that MST has the same clinical potential as ECT and can almost ignore cognitive side effects [7, 8]. However, the mechanism of MST protecting cognition is still unknown. Compared with ECT, the studies of MST treating major depression recorded shorter reorientation times after treatment [9] and fewer cognitive side effects [10]. As an alternative to ECT, the reduced cognitive side effects of MST illustrate a unique mechanism of action. For example, MST provides more targeted stimuli [11], while field modeling studies show that ECT stimulates a wide range of cortical and subcortical areas, including structures related to cognitive function, such as the medial temporal lobe [6].

Cortical thinning is a well‐recognized feature of schizophrenia and can be found at all stages of schizophrenia [12]. Cortical thinning can potentially predict the risk for psychosis among first‐degree unaffected adolescent relatives of schizophrenia patients [13]. Cortical thinning is one of the most common findings associated with cognitive impairment in schizophrenia [14, 15]. Up to now, no study has investigated the effect of ECT on the cortical thickness of schizophrenic patients. However, at least three studies have found that ECT can increase the cortical thickness of patients with depression. Study 1 found that bilateral ECT induced a bilateral cortical thickness increase, including the temporal pole, infratemporal and middle temporal cortex, and insula [16]. A longitudinal MRI study found that the cortical thickness of 26 regions after ECT in patients with major depression increased significantly, but the increase was transient [17]. Another study on the treatment of major depression with ECT found that the increase occurred in the temporal cortex (basically temporal pole and insula) [18].

The development of modern MRI technology makes it possible to quantitatively measure the anatomical characteristics of the brain. So far, no study has investigated the effect of MST on cortical thickness in schizophrenic patients. To solve this problem, we use the FreeSurfer image analysis suite (http://surfer.nmr.mgh.harvard.edu/fswiki/FreeSurferWiki). To explore the MRI and cognitive scale data sets of schizophrenics treated with ECT or MST to calculate the cortical thickness, psychopathology, cognitive score, and the relationship between them. This study is helpful in further understanding the influence of MST treatment on the cortical thickness of schizophrenics and the relationship between the changes in cortical thickness and psychopathology and cognitive function after MST treatment.

## 2 Materials and Methods

### 2.1 Sample

Thirty‐four patients with schizophrenia (16 ECT and 18 MST) were included in this study, as described in our previous work [19]. This study was approved by the Ethics Committee of Shanghai Mental Health Center (No. 2014‐30R). Patients who are interested in this study and meet the inclusion/exclusion requirements have provided informed written consent. The MST equipment used in this study is currently undergoing maintenance, and the study cannot continue until additional maintenance funding is obtained.

Inclusion criteria were as follows: (1) between the ages of 18–55; (2) a diagnosis of schizophrenia based on the Diagnostic and Statistical Manual of Mental Disorders, Fifth Edition (DSM‐5); (3) clinical indications for convulsive therapy, such as severe psychomotor excitement or retardation, suicide attempt, highly aggressive behavior, pharmacotherapy intolerance, and (or) ineffectiveness of antipsychotics [20] as assessed by two attending doctors; (4) Positive and Negative Syndrome Scale (PANSS) score ≥ 60; and (5) patients with other mental issues, serious actual illnesses, giving research facility irregularities, inability to answer a sufficient preliminary of ECT, or pregnant or expecting to become pregnant during the review were prohibited.

Using a random number sequence that was generated by SAS 9.3 (SAS Institute Inc., USA), patients were divided into treatment groups in a ratio of one to one. An independent biostatistician created the sequence without knowing anything about the subjects of the study. Each subject received a number inside an envelope showing a randomization task. Before the first ECT or MST session, the treating clinician received the treatment code after baseline assessments. All methodologies before treatment and the room arrangement were indistinguishable (counting the presence of both ECT and MST gear) to guarantee patient blinding. Additionally, a trained psychiatrist who was not aware of the treatment group assignment conducted clinical and cognitive assessments.

### 2.2 MST and ECT Procedures

The setting for ECT and MST was similar to that of typical Chinese clinical practice [21]. The participants were scheduled for 10 sessions of MST or ECT over the course of 4 weeks, with three sessions per week for the first 2 weeks and two sessions per week for the following 2 weeks. This was in addition to the usual treatment. Both MST and ECT were controlled under broad sedation with intravenous etomidate (0.21–0.3 mg/kg) and propofol (1.82–2.44 mg/kg). In addition, succinylcholine (1 mg/kg) and atropine (0.5 mg) were given to patients intravenously to lessen airway secretion and relax their muscles.

A MagPro X100 from MagVenture A/S, Denmark, was used to administer the MST at 25 Hz, 100% output, and 370 s pulse width. The magnetic field had a peak intensity of 4.2 Tesla. Considering that the seizure limit is probably going to increment as treatment continues [22], a titration strategy was utilized to decide the base compelling term of attractive feeling. The feeling started at 4 s and was expanded on each ensuing meeting by 4 s to a limit of 20 s (i.e., 100 heartbeats to 500 heartbeats for every meeting). On the off chance that seizure quality was poor (length of < 15 s) in a given meeting, the term was expanded by 8 s for the following meeting. If no seizure occurred, an additional 20‐s stimulation was administered right away. A twin coil with its midline on the vertex was used to deliver magnetic stimulation, and it was found to produce a stronger and deeper electric field than other MST coil configurations [6].

The Thymatron System IV device from Somatics, USA was used to administer bitemporal ECT. The pulse width was set to 1.0 ms, and the intensity (percent energy) was automatically calculated using the default setting (0.8 times age).

Using frontal and mastoid electrodes, the electroencephalogram (EEG) was recorded from the prefrontal cortex. From the beginning of the magnetic or electric stimulation to the end, the duration of the seizures was measured.

### 2.3 Clinical and Cognitive Assessment

Form A of the Repeatable Battery for the Assessment of Neuropsychological Status (RBANS) was given at the beginning of the study, and Form B was given at the end of the study. These tests were used to measure baseline cognition as well as improvements (or negative effects) brought about by treatment. The Positive and Negative Syndrome Scale (PANSS) was used to measure how severe the symptoms of schizophrenia were. The rater was not aware of the patient group assignment for any of the psychometric scales.

### 2.4 Magnetic Resonance Image Acquisition

Structural MRI scans were given to patients 24 h before the first session (T1, pre‐ECT, or pre‐MST) and 24–48 h after the last session (T2, post‐ECT, or post‐MST). Subjects were asked to close their eyes but remain awake and not concentrate on anything specific during the scans. Pictures were gained utilizing a 3‐Tesla scanner (Siemens verio syngo MR B17) with a 32‐channel head coil. To stop the head from moving, foam pads were used to hold it in place. A 3D T1‐weighted magnetization‐prepared rapid gradient echo (MPRAGE) sequence was used to collect all of the data (TR = 2530 ms, TE = 2.56 ms, field of view = 256 × 256 mm^2^, matrix size = 256 × 256, flip angle = 7°, slice thickness = 1.0 mm, 224 contiguous slices, voxel size = 1.0 × 1.0 × 1.0 mm^3^).

### 2.5 Image Preprocessing

All picture preprocessing steps were led using the REST_1.8 tool compartment (http://restfmri.net/discussion/index.php?q=rest) and SPM12 programming bundle (Measurable Parametric Planning programming: www.fil.ion.ucl.ac.uk/spm) in Matlab (MathWorks, Natick, MA), 2011b First, the images were visually inspected by the experienced member (Jin Li) for the presence of any artifacts that would prevent further analyses. Then, the 3D‐MPRAGE series of images was manually reoriented to the anterior–posterior commissure (AC‐PC) line.

FreeSurfer 7.2.0 (http://surfer.nmr.harvard.edu/fswiki/FreeSurferWiki) was used by us to process the magnetic resonance images. edu/). To reconstruct the cortical surface, FreeSurfer's standard auto‐reconstruction algorithm was used to calculate the cortical thickness. An experienced investigator carefully examined each reconstructed image. Using a 30‐mm Gaussian kernel, each sample was projected onto the average and smoothed.

### 2.6 Statistical Analysis

IBM SPSS Statistics 26 was used for all statistical analyses. To evaluate the normality of the data, we employed histogram analysis. By plotting the data into bins, we can visualize the distribution's shape, which can provide insights into its normality. In this study, all continuous variables except gender were found to follow a normal distribution. The independent samples *t*‐test or the *χ* ^2^ test, as appropriate, were used to compare the patient characteristics of the ECT and MST groups. Repeated measures analysis of variance (RT‐ANOVA) was used to compare clinical symptoms and cognitive performance before and after treatment.

Cortical thickness differences before and after MST or ECT treatment were compared using FreeSurfer's general linear model. We utilized a permutation test (Monte Carlo simulation) to correct for multiple comparisons. Improved PANSS symptoms and cognitive scores are correlated with changes in cortical thickness, with age and education level serving as control variables. The MST and ECT groups were the subjects of correlation analysis, respectively. We used Bonferroni correction for multiple comparisons and Spearman's rho to determine the associations between cortical thickness, neurocognitive assessment scores, and symptom measures. A 0.05 *p*‐value was used.

## 3 Results

### 3.1 Demographics and Clinical Characteristics

Demographic and clinical manifestations are shown in Table 1. The MST group had a longer illness duration (*p* = 0.049), but there were no significant differences in the sex ratio, mean age, educational level, duration of the current episode, or daily chlorpromazine equivalent dose between the groups. Each participant in this study successfully induced seizures in all sessions. The average EEG duration of the MST group was 13.9 ± 3.9 s, while that of the ECT group was 33.8 ± 6 s.

**TABLE 1:** Demographic, clinical characteristics, and neuropsychological scores in the ECT and MST groups.

	ECT (*n* = 16)	MST (*n* = 18)	*F/t/χ* ^2^			*p* (treatment, time, treatment × time)		
Sex (female/male)^a^	6/10	9/9		0.537			0.510	
Age (years)	30.9 (11.2)	32.1 (11.3)		0.001			0.973	
Education (years)	10.9 (3.1)	12.5 (3.6)		0.010			0.923	
Duration of illness (months)	61.8 (44.5)	99.2 (74.9)		4.195			0.049*;	
Duration of current episode (days)	53.4 (53.2)	62.3 (92.6)		1.507			0.229	
CPZE (mg)	397.1 (266.9)	501.1 (244.2)		0.264			0.611	
PANSS_Total (T0)	92.7 (15.9)	95.3 (9.6)	0.096	99.646	0.276	0.759	0.000*	0.603
PANSS_Total (T1)	68.2 (14.9)	68.1 (15.9)						
Positive score (T0)	26.2 (6.5)	26.5 (4.8)	0.158	89.173	0.134	0.694	0.000*	0.717
Positive score (T1)	15.6 (5.1)	16.7 (7.5)						
Negative score (T0)	20.9 (6.1)	22.3 (6.5)	0.007	15.033	2.163	0.935	0.000*	0.151
Negative score (T1)	18.8 (5.7)	17.6 (6.3)						
General score (T0)	45.6 (9.3)	46.5 (4.7)	0.039	88.353	0.125	0.844	0.000*	0.726
General score (T1)	33.9 (7.4)	33.8 (7.0)						
RBANS_Total (T0)	76.9 (13.1)	76.2 (10.5)	0.223	5.230	2.768	0.640	0.029*	0.106
RBANS_Total (T1)	71.1 (13.6)	75.3 (8.2)						
Immediate memory (T0)	75.2 (19.6)	69.4 (14.1)	0.156	0.006	6.936	0.696	0.936	0.013*
Immediate memory (T1)	71.5 (21.5)	72.8 (12.6)						
Visuospatial/constructional (T0)	87.2 (13.1)	86.2 (13.0)	0.150	9.982	0.060	0.701	0.003*	0.808
Visuospatial/constructional (T1)	81.9 (12.4)	79.9 (10.9)						
Language score (T0)	79.2 (6.1)	82.1 (12.0)	7.746	12.211	6.052	0.009*	0.001*	0.019*
Language score (T1)	81.5 (12.1)	94.0 (7.7)						
Attention score (T0)	95.2 (11.0)	97.2 (9.1)	0.465	34.250	0.023	0.500	0.000*	0.881
Attention score (T1)	87.9 (9.7)	90.3 (10.5)						
Delayed memory (T0)	74.8 (17.8)	71.7 (12.7)	0.199	14.226	3.569	0.659	0.001*	0.068
Delayed memory (T1)	59.8 (15.5)	66.7 (13.4)						

*Note:* Our previous work serves as the basis for this table [19]. Data are displayed as mean ± standard deviation.

Abbreviations: CPZE, daily chlorpromazine equivalent dose; ECT, electroconvulsive therapy; MST, magnetic seizure therapy.

^a^ *p* value obtained by the chi‐squared test.

* *p* < 0.05.

On the PANSS total score or subscale scores, there was neither a main effect of the group (ECT vs. MST) nor an interaction effect of the group × time (pre‐treatment T1 vs. post‐treatment T2). As a result, the PANSS score was lower (symptoms improved) with both treatments without a significant difference in efficacy between the groups.

The group × time interaction had a significant impact on the RBANS language score (*p* = 0.019). There were additionally significant main effects of group (*p* = 0.009) and time (*p* = 0.001) on the RBANS language score, and simple effects analysis uncovered that MST significantly expanded the language score, while the impact of ECT did not reach significance.

### 3.2 Comparison of Cortical Thickness

Neither the ECT nor the MST groups showed any significant changes in cortical thickness before or after treatment, according to our analysis.

## 4 Discussion

In this study, we investigated the MRI, clinical improvement, and cognitive scale datasets of schizophrenia patients receiving ECT or MST treatment. We used the FreeSurfer image analysis kit (http://surfer.nmr.mgh.harvard.edu/fswiki/FreeSurferWiki) to calculate the changes in cortical thickness before and after treatment. We plan to explore the relationship between changes in cortical thickness, treatment effectiveness, and cognitive changes. After the comparison of cortical thickness changes in FreeSurfer's General linear model and Monte Carlo simulation multiple comparison correction, no significant cortical thickness changes were found in both groups. So we suspended the relevant analysis in the plan.

Cortical thinning is a well‐known characteristic of schizophrenia and can occur at any stage [12]. No research has yet looked at how ECT or MST affects schizophrenia individuals' cortical thickness. The reduced cognitive side effects of MST as an alternative to ECT demonstrate a unique mode of action. For instance, field modeling studies indicate that MST delivers more focused stimuli [11], whereas ECT activates a variety of cortical and subcortical regions, including those involved in cognitive function, such as the medial temporal lobe [6].

However, research on the treatment of schizophrenia with MST is still in its infancy. MST is one of the new non‐invasive brain stimulation technologies being studied in recent years. Due to a lack of data, no conclusions can be drawn on the efficacy and tolerability of MST in patients with schizophrenia [22]. The carefully designed RCT has reason to answer this question. Based on existing knowledge, this study investigates for the first time the effect of MST on cortical thickness in patients with schizophrenia. We found that MST had no effect on cortical thickness in patients with schizophrenia.

Given the absence of a healthy control group in this study, it is not feasible to ascertain whether the cortical thickness of patients with schizophrenia in this cohort is diminished compared to that of healthy individuals. Moreover, the short intervention duration (4 weeks) has resulted in the effects of MST or ECT on cortical thickness not yet being manifested. Additionally, the relatively small sample size restricts the statistical power to detect a significant impact of MST or ECT on cortical thickness.

Our study found that the cognitive side effects of ECT are more pronounced, and MST has a cognitive retention effect. Regarding the mechanism of its cognitive preservation effect, our previous articles have explored that it does not affect the volume of hippocampal substructures and is related to its cognitive preservation mechanism [19]. This study found that MST does not affect the cortical thickness of patients and can serve as one of the directions for exploring the mechanism of MST cognitive preservation in the future.

Language disorder is a marker for schizophrenia [23] and can also serve as a biomarker for schizophrenia [24]. In this study, patients with schizophrenia showed a significant increase in language scores after MST treatment. However, there was no impairment or increase in language scores in the group receiving ECT treatment. These indicate that MST not only has cognitive protective effects but also has special advantages in improving language function. Unfortunately, we did not find a significant correlation (*p* = 0.808) between improved language performance scores and decreased PANSS scores in the MST group. As mentioned earlier, our previous article suggests that MST does not affect the volume of hippocampal substructures, which may be related to its cognitive protective mechanisms [19]. We speculate that the significant improvement in language by MST may be related to its enhanced functional connectivity between the left inferior frontal gyrus and the anterior cingulate cortex [25], although further verification is needed.

It is generally believed that declarative memory is significantly impaired after ECT, but immediate memory is widely preserved [26]. The interaction effect (time × group) in this study was statistically significant, indicating that the trend of changes in immediate memory scores varies, but the group effect is not substantial. Immediate memory impairment is not only seen in individuals with schizophrenia but also in their non‐affected relatives [27]. We have reason to believe that MST has special advantages in improving immediate memory in patients with schizophrenia. We did not find a significant correlation (*p* = 0.492) between the immediate memory improvement score and the PANSS reduction score in the MST group. A study reported a correlation between immediate memory and serum cholesterol levels in patients with schizophrenia [28].

There are a number of limits to this study. First, there are still questions regarding the precision of machine‐automated segmentation; however, it has benefits in that it is standardized, unbiased, and easy to reproduce. Second, because the patients in this study had been prescribed antipsychotics, we were unable to rule out an effect of these on brain morphological characteristics. In the future, novel design plans and the implementation of novel methods will help to solve this problem. Third, in accordance with the rising regard for the ecological validity [29] of the neuropsychological appraisal, the low biological legitimacy of the executive functions test is one of the impediments to this examination. Fourth, randomized clinical trials have consistently shown that right unilateral (RUL) ECT is associated with superior cognitive outcomes compared to bilateral ECT [30, 31, 32, 33]. In future research, we will investigate the effects of MST and RUL ECT on cognitive function in patients with schizophrenia. Finally, Exploring the effect of MST on cortical thickness in patients with schizophrenia may require longer intervention and follow‐up periods. In future research, we will optimize the intervention paradigm and conduct long‐term follow‐up.

ECT is one of the most effective methods for treating severe mental illnesses, but it can also lead to some of the most serious side effects. Therefore, determining the method of brain stimulation that matches the clinical efficacy of ECT without any side effects is an important goal in psychiatry. We investigated the differences between ECT and MST in clinical samples of patients with schizophrenia. Our research shows that MST achieved the same PANSS reduction as ECT, while showing fewer side effects in terms of RBANS changes. We examined the structural changes through MRI and found that neither treatment significantly altered cortical thickness.

## Author Contributions

Jin Li and Junjie Wang recruited subjects, collected clinical data, performed schizophrenia symptom assessments, analyzed data, and wrote the manuscript. Yong Yang and Ju Gao conducted clinical and cognitive symptom assessments. Xiaobin Zhang designed the study and prepared the manuscript. All authors participated in the preparation of the manuscript and approved its final version.

## Conflicts of Interest

The authors declare no conflicts of interest.

## Data Availability

The datasets generated and/or analyzed during the current study are available from the corresponding author upon reasonable request.
